# 3D shape reconstruction with a multiple-constraint estimation approach

**DOI:** 10.3389/fnins.2023.1191574

**Published:** 2023-05-19

**Authors:** Xia Chen, Zhan-Li Sun, Ying Zhang

**Affiliations:** ^1^School of Information and Computer, Anhui Agricultural University, Hefei, China; ^2^Key Laboratory of Intelligent Computing and Signal Processing of Ministry of Education, Institute of Physical Science and Information Technology, Anhui University, Hefei, China; ^3^Anhui Provincial Key Laboratory of Multimodal Cognitive Computation, Anhui University, Hefei, China; ^4^School of Electrical Engineering and Automation, Anhui University, Hefei, China; ^5^Information Materials and Intelligent Sensing Laboratory of Anhui Province, Anhui University, Hefei, China

**Keywords:** non-rigid structure from motion, elastic net, similarity constraint, Augmented Lagrange multipliers, 3D reconstruction

## Abstract

In this study, a multiple-constraint estimation algorithm is presented to estimate the 3D shape of a 2D image sequence. Given the training data, a sparse representation model with an elastic net, i.e., *l*_1_−norm and *l*_2_−norm constraints, is devised to extract the shape bases. In the sparse model, the *l*_1_−norm and *l*_2_−norm constraints are enforced to regulate the sparsity and scale of coefficients, respectively. After obtaining the shape bases, a penalized least-square model is formulated to estimate 3D shape and motion, by considering the orthogonal constraint of the transformation matrix, and the similarity constraint between the 2D observations and the shape bases. Moreover, an Augmented Lagrange Multipliers (ALM) iterative algorithm is adopted to solve the optimization of the proposed approach. Experimental results on the well-known CMU image sequences demonstrate the effectiveness and feasibility of the proposed model.

## 1. Introduction

As an important component of computer vision, 3D shape reconstruction has been widely used in many applications (Li et al., 2016, 2018; Adamkiewicz et al., 2022; Chiang et al., 2022; Fombona-Pascual et al., 2022; Jang et al., 2022; Lu et al., 2022; Nian et al., 2022a,b; Wang et al., 2022; Wen et al., 2022). Among the various 3D shape reconstruction methods, non-rigid structure from motion (NRSFM) offers a technique to simultaneously recover the 3D structures and motions of an object, by using the 2D landmarks in a series of images (Graßhof and Brandt, 2022; Kumar and Van Gool, 2022; Song et al., 2022). Nevertheless, NRSFM is still an underconstrained and challenging issue because of lacking any prior knowledge of 3D structure deformation.

To alleviate the uncertainty, the various constraints are exploited constantly. Bregler et al. (2000), proposed a low-rank constraint-based approach to decompose the observation matrix into a motion factor and a shape basis. In order to reduce the number of the unknown variables proposed by Bregler et al. (2000), a point trajectory approach was presented by Akhter et al. (2010) by using the predefined bases of discrete cosine transform (DCT). However, the high-frequency deformation cannot be reconstructed well via this trajectory representation because of the low-rank constraint. Gotardo and Martinez (2011) modeled a smoothly deforming 3D shape as a single point moving along a smooth time trajectory within a linear shape space. In addition to the low-rank constraint, the higher frequency DCT was adopted to capture the high-frequency deformation.

For the low-rank constraint methods, it is difficult to determine the optimal number of shape bases or trajectory bases. To solve this problem, a Procrustean normal distribution (PND) model was presented by Lee et al. (2013) to separate the motion and deformation components strictly, without any additional constraints or prior knowledge. The experimental results demonstrate the performance of PND. Subsequently, the Procrustean Markov Process (PMP) algorithm was proposed by Lee et al. (2014), by combing in a first-order Markov model representing the smoothness between two adjacent frames with PND. Lee et al. (2016) reported a consensus of non-rigid reconstruction (CNR) approach to estimate 3D shapes based on local patches. However, the reconstruction performance of these methods may degrade significantly when the number of images becomes small, especially for a single image.

Referring to the active shape model (Cootes et al., 1995), a limb length constraint-based approach was presented by Wang et al. (2014) to estimate the 3D shape of an object from a single 2D image, by solving a *l*_1_−norm minimization problem. Zhou et al. (2013) proposed a sparse representation-based convex relaxation approach (CRA) to guarantee global optimality. The shape bases were extracted from a given training data by using a sparse representation model. The corresponding coefficients were obtained by adopting a convex relaxation assumption. A prominent advantage of CRA is that the algorithm can deal with a single image.

To further enhance the performance of the CRA algorithm, a multiple-constraint-based estimation approach is proposed to estimate the 3D shape of a 2D image sequence. Inspired by Zhang and Xing (2017), a dictionary learning model with *l*_1_−norm and *l*_2_−norm, i.e., elastic net, is constructed to extract more effective shape bases from a given training set. Referring to (Cheng et al., 2015), a penalized least-square model is constructed to estimate 3D shape and motion, by considering the orthogonal constraint of the transformation matrix and the similarity constraint between the 2D observations and the shape bases. In addition, an augmented Lagrange multipliers (ALM) iterative algorithm is developed to optimize the reconstruction model. The effectiveness and feasibility of the proposed algorithm are verified on the well-known CMU image sequences.

The rest of this article is organized as follows. A detailed description of the designed MCM-RR approach is introduced in Section 2. In Section 3, we report the experimental results. Finally, the article is concluded in Section 4.

## 2. Methods

According to the shape-space model by Zhou et al. (2013), the unknown 3D shape **S** ∈ ℝ^3×*p*^ is constructed as a linear combination of a few shape bases ${\text{B}}_{i}\in {\mathbb{R}}^{3\times p}$, i.e.,


(1) $$\begin{array}{l} \text{S}=\overset{K}{\underset{i=1}{\sum }}c_{i}{\text{R}}_{i}{\text{B}}_{i}, \end{array}$$

where *p* and *K* are the numbers of feature points and shape bases, respectively. The parameter *c*_*i*_ and ${\text{R}}_{i}\in {\mathbb{R}}^{3\times 3}$ denote the coefficient and rotation matrix, respectively. In terms of the weak-perspective projection model, the corresponding 2D observations are modeled as a matrix **W** ∈ ℝ^2×*p*^,


(2) $$\begin{array}{l} \text{W}=\overset{K}{\underset{i=1}{\sum }}{\text{M}}_{i}{\text{B}}_{i}. \end{array}$$

The matrix ${\text{M}}_{i}\in {\mathbb{R}}^{2\times 3}$ can be represented as


(3) $$\begin{array}{l} {\text{M}}_{i}=c_{i}{\tilde{R}}_{i}, \end{array}$$

where ${\tilde{R}}_{i}\in {\mathbb{R}}^{2\times 3}$ is the first two rows of **R**_*i*_. Combining the orthogonal constraint, the matrix **M**_*i*_ satisfies


(4) $$\begin{array}{l} {\text{M}}_{i}{\text{M}}_{i}^{T}=c_{i}^{2}{\text{I}}_{2}, \end{array}$$

where ${\text{I}}_{2}\in {\mathbb{R}}^{2\times 2}$ is an identity matrix. The 3D shape, i.e., *z*−coordinates, and the motion parameters *c*_*i*_ and **R**_*i*_, are estimated by utilizing the observations **W**, i.e., the (*x, y*) coordinates of feature points.

In the proposed method, the shape bases **B** ∈ ℝ^3*K*×*p*^ are extracted via a sparse model with the elastic net constraint. The **B** is the stacking of **B**_*i*_(*i* = 1, ..., *K*). The matrix **M** are solved by a penalized least-square model. Given **M**, the parameters *c*_*i*_ and **R**_*i*_ are derived via refinement decompose (Zhou et al., 2013). After obtaining *c*_*i*_, **R**_*i*_ and **B**_*i*_, the unknown 3D shape can be computed via (1). The pseudocode of the proposed algorithm is summarized in 1. The pseudocode of the proposed algorithm is summarized in Algorithm 1.

**Algorithm 1:** Pseudocode of the MCM-RR algorithm.

1: Compute the shape bases **B** via the elastic net based sparse model (5).
2: Initialize α, β, γ.
3: Initialize **M**^0^, **Z**^0^, **Y**^0^, μ^0^, *t* = 0.
4: **while** *t* < = 1000 **do**
5: Compute the optimized **M**^*t*+1^ according to (15) by fixing **Z**^*t*^, **Y**^*t*^, and μ^*t*^,
6: Update **Z**^*t*+1^ via (17) by fixing **M**^*t*+1^, **Y**^*t*^, and μ^*t*^,
7: Update **Y**^*t*+1^ via (18) by fixing **M**^*t*+1^, **Z**^*t*+1^,
8: **if** δ_1_ < ε* & δ*_2_ < ε **then**
9: break,
10: **else**
11: **if** δ_1_ > 10δ_2_ **then**
12: μ^*t*+1^ = 2μ^*t*^,
13: **else** {δ_2_ > 10δ_1_}
14: μ^*t*+1^ = μ^*t*^/2.
15: **end if**
16: **end if**
17: Update *t* ← *t* + 1.
18: **end while**
19: **if** refinement reconstruction **then**
20: Compute **R** and **c** according to (22) via the alternating minimization (Zhou et al., 2013).
21: **end if**
22: Estimate **S** by using (1)

### 2.1. Extraction of shape bases via a sparse model with elastic net constraint

For a given 3D training set **A** ∈ ℝ^3*p*×*F*^, i.e., the (*x, y, z*) coordinates of feature points of training images, the shape bases **N** ∈ ℝ^3*p*×*K*^ and the coefficient matrix **X** ∈ ℝ^*K*×*F*^ can be obtained from the following sparse model:


(5) $$\begin{array}{l} \begin{array}{c} \underset{{\text{N}}_{1},\cdots \;,{\text{N}}_{K}}{\text{min}}\;\frac{1}{2}{\left\|\text{A}-\text{N}\text{X}\right\|}_{F}^{2}+\lambda \left(\tau {\left\|\text{X}\right\|}_{1}+\left(1-\tau \right){\left\|\text{X}\right\|}_{2}^{2}\right)\\ \text{s.t.}{\left\|{\text{N}}_{i}\right\|}_{F}\leq 1,X_{ij}\geq 0,\forall i\in \left[1,K\right],j\in \left[1,F\right], \end{array} \end{array}$$

where *F* and τ are the number of frames and a weight coefficient, respectively. The ${\text{N}}_{i}\in {\mathbb{R}}^{3p\times 1}$ is the *i*-th column of **N**. The linear combination of *l*_1_−norm and *l*_2_−norm, called elastic net constraint, are enforced to constraint the sparsity of coefficients **X** as well as scale. The parameter λ is a trade-off parameter between the reconstruction error and the elastic net constraint.

For (5), we first compute the partial differentials of **X** and **N**, i.e.,


(6) $$\begin{array}{l} \partial \text{X}={\left({\text{N}}^{t}\right)}^{T}\left(\text{A}-{\text{N}}^{t}\text{X}\right)+\lambda \left(\tau {\text{I}}_{KF}+2\left(1-\tau \right)\text{X}\right), \end{array}$$

(7) $$\begin{array}{l} \partial \text{N}=\left(\text{A}-\text{N}{\left({\text{X}}^{t+1}\right)}^{T}\right){\left({\text{X}}^{t+1}\right)}^{T}, \end{array}$$

where **I**_*KF*_ is a *K* × *F* identity matrix. Thereafter, **X** and **N** can be updated alternately as


(8) $$\begin{array}{l} {\text{X}}^{t+1}={\text{X}}^{t}-\phi_{1}\partial \text{X}, \end{array}$$

(9) $$\begin{array}{l} {\text{N}}^{t+1}={\text{N}}^{t}-\phi_{2}\partial \text{N}, \end{array}$$

where ϕ_1_ and ϕ_2_ are the step size of ∂**X** and ∂**N**, respectively. After convergence, the shape bases **B** can be obtained by a re-arrangement of **N**.

### 2.2. 3D shape estimation via a penalized least-square model with similarity constraint

In terms of (2), the proposed penalized least-square model, including a relaxed orthogonality constraint (Zhou et al., 2013) and a similarity constraint (Cheng et al., 2015) can be formulated as


(10) $$\begin{array}{l} \begin{array}{cc} \underset{\tilde{\text{M}},\text{Z}}{\text{min}} & \;\frac{1}{2}{\left\|\text{W}-\text{Z}\tilde{\text{B}}\right\|}_{F}^{2}+\alpha \overset{K}{\underset{i=1}{\sum }}{\left\|{\text{M}}_{i}\right\|}_{2}+\frac{\beta }{2}{\left\|\text{Z}\text{D}\right\|}_{2}^{2}\\ \text{s.t.} & \;\tilde{\text{M}}=\text{Z}, \end{array} \end{array}$$

where **Z** ∈ ℝ^2×3*K*^ is an auxiliary variable and $\tilde{\text{M}}=\left[{\text{M}}_{1},\cdots \;,{\text{M}}_{K}\right]$, $\tilde{\text{B}}={\left[{\text{B}}_{1}^{T},\cdots \;,{\text{B}}_{K}^{T}\right]}^{T}$. The parameters α and β are used to weight the two regularization terms. The diagonal matrix **D** ∈ ℝ^3*K*×3*K*^ is represented as


(11) $$\begin{array}{l} \text{D}=\left(\bar{\text{D}}⊗{\text{I}}_{3}\right). \end{array}$$

For the diagonal similarity matrix $\bar{\text{D}}\in {\mathbb{R}}^{K\times K}$, the diagonal element *d*_*i*_ is computed as


(12) $$\begin{array}{l} d_{i}=\exp\left(\frac{\left\|\text{W}-\Pi {\text{B}}_{i}\right\|}{2\gamma^{2}}\right), \end{array}$$

where Π = [1, 0, 0;0, 1, 0], γ^2^ is the parameter of an exponential function.

With the ALM iterative algorithm, the penalized least-square model (10) can be reformulated as


(13) $$\begin{array}{l} \begin{array}{c} L=\frac{1}{2}{\left\|\text{W}-\text{Z}\tilde{\text{B}}\right\|}_{F}^{2}+\alpha \overset{K}{\underset{i=1}{\sum }}{\left\|{\text{M}}_{i}\right\|}_{2}+\langle \text{Y},\tilde{\text{M}}-\text{Z}\rangle \\ +\frac{\beta }{2}{\left\|\text{Z}\text{D}\right\|}_{2}^{2}+\frac{\mu }{2}{\left\|\tilde{\text{M}}-\text{Z}\right\|}_{F}^{2}, \end{array} \end{array}$$

where **Y** and μ are a dual variable and a weight of penalty term, respectively. In (13), there are four unknown variables $\tilde{\text{M}}$, **Z**, **Y**, and μ. The solutions can be solved by the alternating direction method of multipliers (ADMM).

First, the optimal $\tilde{\text{M}}$ at the (*t* + 1)^*th*^ iteration can be formulated as


(14) $$\begin{array}{l} {\tilde{\text{M}}}^{t+1}=\text{arg }\underset{\tilde{\text{M}}}{\text{min}}\overset{K}{\underset{i=1}{\sum }}\frac{1}{2}{\left\|{\text{M}}_{i}-{\text{P}}_{i}^{t}\right\|}_{F}^{2}+\frac{\alpha }{\mu }{\left\|{\text{M}}_{i}\right\|}_{2}, \end{array}$$

where ${\text{P}}_{i}^{t}$ is the *i*^*th*^ column-triple of ${\text{Z}}^{t}-\frac{1}{\mu }{\text{Y}}^{t}$. According to the proximal problem (Zhou et al., 2013), ${\text{M}}_{i}^{t+1}$ can be computed as


(15) $$\begin{array}{l} {\text{M}}_{i}^{t+1}=U\text{diag}\left(\Sigma -\frac{\alpha }{\mu }P_{l_{1}}\left(\frac{\Sigma \mu }{\alpha }\right)\right)V^{T},i\in \left[1,K\right], \end{array}$$

where $U\Sigma V^{T}=svd\left({\text{P}}_{i}^{t}\right)$. The operation $P_{l_{1}}\left(\cdot \right)$ denotes the projection of a vector to the unit *l*_1_−norm ball (Zhou et al., 2013).

Similarity, the optimal **Z** at the (*t* + 1)^*th*^ iteration can be formulated as


(16) $$\begin{array}{l} \begin{array}{c} {\text{Z}}^{t+1}=\text{arg }\underset{\text{Z}}{\text{min}}\frac{1}{2}{\left\|\text{W}-\text{Z}\tilde{\text{B}}\right\|}_{F}^{2}+\langle {\text{Y}}^{t},{\tilde{\text{M}}}^{t+1}-\text{Z}\rangle \\ +\frac{\beta }{2}{\left\|\text{Z}\text{D}\right\|}_{2}^{2}+\frac{\mu }{2}{\left\|{\tilde{\text{M}}}^{t+1}-\text{Z}\right\|}_{F}^{2}. \end{array} \end{array}$$

We compute the one-order partial derivative of (16) with respect to **Z** and set it as zero. Thereafter, **Z**^*t*+1^ can be given by


(17) $$\begin{array}{l} {\text{Z}}^{t+1}=\left(\text{W}{\tilde{\text{B}}}^{T}+\mu {\tilde{\text{M}}}^{t+1}+{\text{Y}}^{t}\right){\left(\tilde{\text{B}}{\tilde{\text{B}}}^{T}+\mu \text{I}+\beta \text{D}{\text{D}}^{T}\right)}^{-1}. \end{array}$$

Afterward, the optimal **Y** at the (*t* + 1)^*th*^ iteration can be computed as


(18) $$\begin{array}{l} {\text{Y}}^{t+1}={\text{Y}}^{t}+\mu \left({\tilde{\text{M}}}^{t+1}-{\text{Z}}^{t+1}\right). \end{array}$$

Given a weight τ, the coefficient μ at the (*t* + 1)^*th*^ iteration can be given by


(19) $$\begin{array}{l} \mu^{t+1}=\{\begin{array}{cc} 2\mu^{t}, & \;if\;\delta_{1}>\tau \delta_{2},\\ \mu^{t}/2, & \;if\;\delta_{2}>\tau \delta_{1}, \end{array} \end{array}$$

where


(20) $$\begin{array}{l} \delta_{1}=\frac{{\left\|{\tilde{\text{M}}}^{t+1}-{\text{Z}}^{t+1}\right\|}_{F}}{{\left\|{\text{Z}}^{t}\right\|}_{F}},\delta_{2}=\frac{{\left\|{\text{Z}}^{t+1}-{\text{Z}}^{t}\right\|}_{F}}{{\left\|{\text{Z}}^{t}\right\|}_{F}}. \end{array}$$

The iterations are repeated until


(21) $$\begin{array}{l} \delta_{1}<\varepsilon \;\&\;\delta_{2}<\varepsilon , \end{array}$$

where ε is a small threshold value. After obtaining **M**_*i*_, the unknown 3D shape can be reconstructed by refinement reconstruction (Zhou et al., 2013).

In the refinement reconstruction, we assume that the rotation matrices of each shape base are equal, denoted as $\bar{\text{R}}$. Thereafter, *c*_*i*_ and $\bar{\text{R}}$ can be estimated by the following rotation synchronization model


(22) $$\begin{array}{l} \begin{array}{c} \underset{\text{c},\bar{\text{R}}}{\text{min}}\;\overset{k}{\underset{i=1}{\sum }}{\left\|{\text{M}}_{i}-c_{i}\bar{\text{R}}\right\|}_{F}^{2}\\ \text{s.t.}\;\bar{\text{R}}{\bar{\text{R}}}^{T}={\text{I}}_{2}, \end{array}, \end{array}$$

which can be solved via the alternating minimization (Zhou et al., 2013). Finally, the 3D shape **S** can be estimated after **M**_*i*_ is obtained.

## 3. Experimental results

### 3.1. Experimental comparison of different algorithms

**Figure 1:**
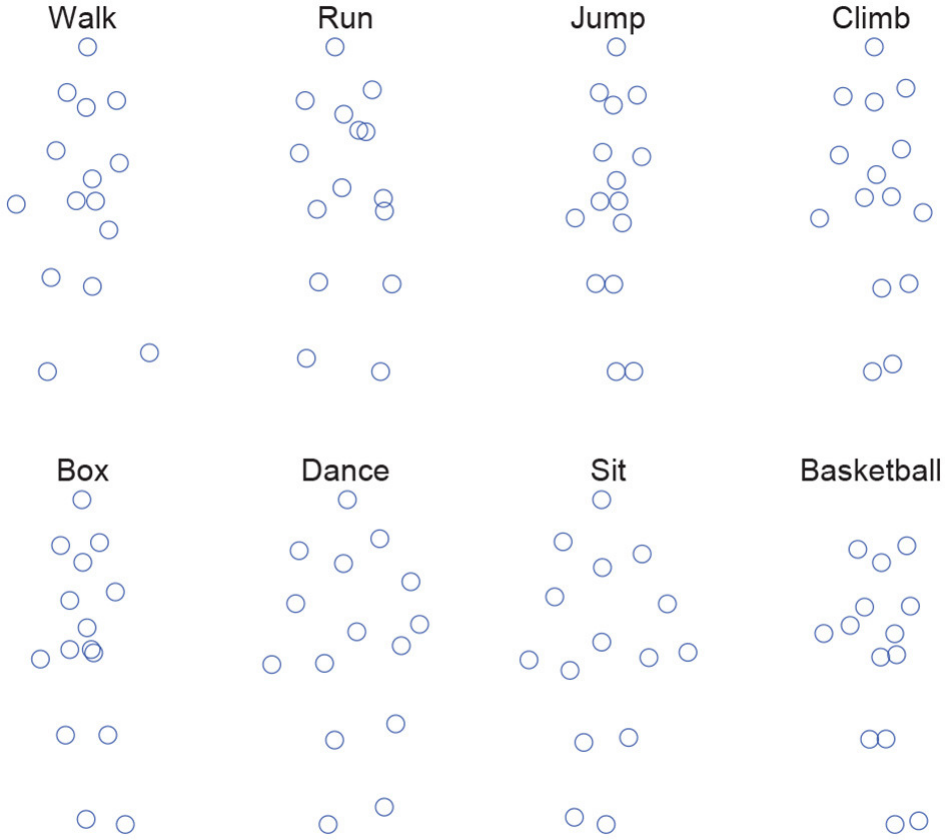
One frame of those eight categories.

The performance evaluation of the proposed 3D shape reconstruction model (denoted as MCM-RR) is carried out on eight motion categories (walk, run, jump, climb, box, dance, sit, and basketball) from the CMU motion capture dataset (Zhou et al., 2013). Figure 1 shows one frame of those eight categories.

In the experiments, the performance of several state-of-the-art 3D shape estimation methods are used to compare with the presented approach, including PND2 (Lee et al., 2013), CNR (Lee et al., 2016), PMP (Lee et al., 2014), and CRA (Zhou et al., 2013).

Mean error ξ of 3D shapes is calculated as the performance indicator to measure the estimation results:


(23) $$\begin{array}{l} \xi =\frac{1}{F}\overset{F}{\underset{t=1}{\sum }}\|{\tilde{\text{S}}}_{t}-{\text{S}}_{t}{\|}_{F}^{2}, \end{array}$$

where ${\tilde{\text{S}}}_{t}\in {\mathbb{R}}^{3\times p}$ and ${\text{S}}_{t}\in {\mathbb{R}}^{3\times p}$ are the reconstructed 3D structure and real 3D structure of *t*^*th*^ frame, respectively.

Table 1 displays the mean and standard deviation (μ±σ) of reconstruction errors ξ of eight motion categories for the five methods, respectively. The best results are highlighted in red, whereas the second best is in blue.

**Table 1:** Mean and standard deviation (μ±σ) of the 3D reconstruction errors ξ of eight motion categories for five methods.

**Sequence**	**PMP**	**CNR**	**PND2**	**CRA**	**MCM-RR**
Walk	97.06 ± 17.35	78.28 ± 15.70	104.20 ± 26.13	38.98 ± 19.64	35.37 ± 18.49
Run	119.37 ± 31.37	65.92 ± 23.69	124.54 ± 28.82	55.69 ± 18.13	52.64 ± 17.05
Jump	102.22 ± 30.74	61.66 ± 40.35	84.64 ± 41.80	57.08 ± 41.56	44.56 ± 27.30
Climb	119.08 ± 39.39	69.36 ± 30.21	87.72 ± 56.04	58.87 ± 24.73	50.25 ± 25.88
Box	252.61 ± 41.28	82.83 ± 33.65	146.91 ± 45.17	72.90 ± 30.64	65.28 ± 26.82
Dance	118.24 ± 35.34	105.73 ± 38.81	118.52 ± 62.07	102.36 ± 44.93	83.59 ± 34.88
Sit	96.31 ± 32.77	69.58 ± 42.18	73.20 ± 32.47	75.68 ± 36.29	62.72 ± 26.79
Basketball	121.26 ± 44.83	67.63 ± 38.97	105.38 ± 72.17	63.66 ± 27.92	57.57 ± 22.96

Table 1 shows the estimation errors of the last two methods are clearly less than that of the first triple algorithms. Among eight categories, the mean reconstruction errors of MCM-RR are the lowest compared to CRA. Moreover, the standard deviations of MCM-RR are less than that of CRA among most categories. Therefore, compared to CRA, both accuracy and robustness are effectively improved for the proposed method.

Compared to CRA, the 3D reconstruction error decreased the percentage ξ_*p*_(%) of MCM-RR can be computed as


(24) $$\begin{array}{c} \xi_{p}=\frac{\xi_{\text{CRA}}-\xi_{\text{MCM-RR}}}{\xi_{\text{CRA}}}\times 100\%. \end{array}$$

From Table 2, we can see that the mean reconstruction errors of MCM-RR decreased about 5.48%∽21.93% compared to CRA. Thus, MCM-RR has a better 3D reconstruction performance than CRA for the eight motion categories.

**Table 2:** Corresponding 3D reconstruction error decreasing percentage ξ_*p*_(%) of MCM-RR compared to CRA for eight motion categories.

**Sequence**	**ξ_*p*_**
Walk	9.26
Run	5.48
Jump	21.93
Climb	14.64
Box	10.43
Dance	18.34
Sit	17.12
Basketball	9.57

**Figure 2:**
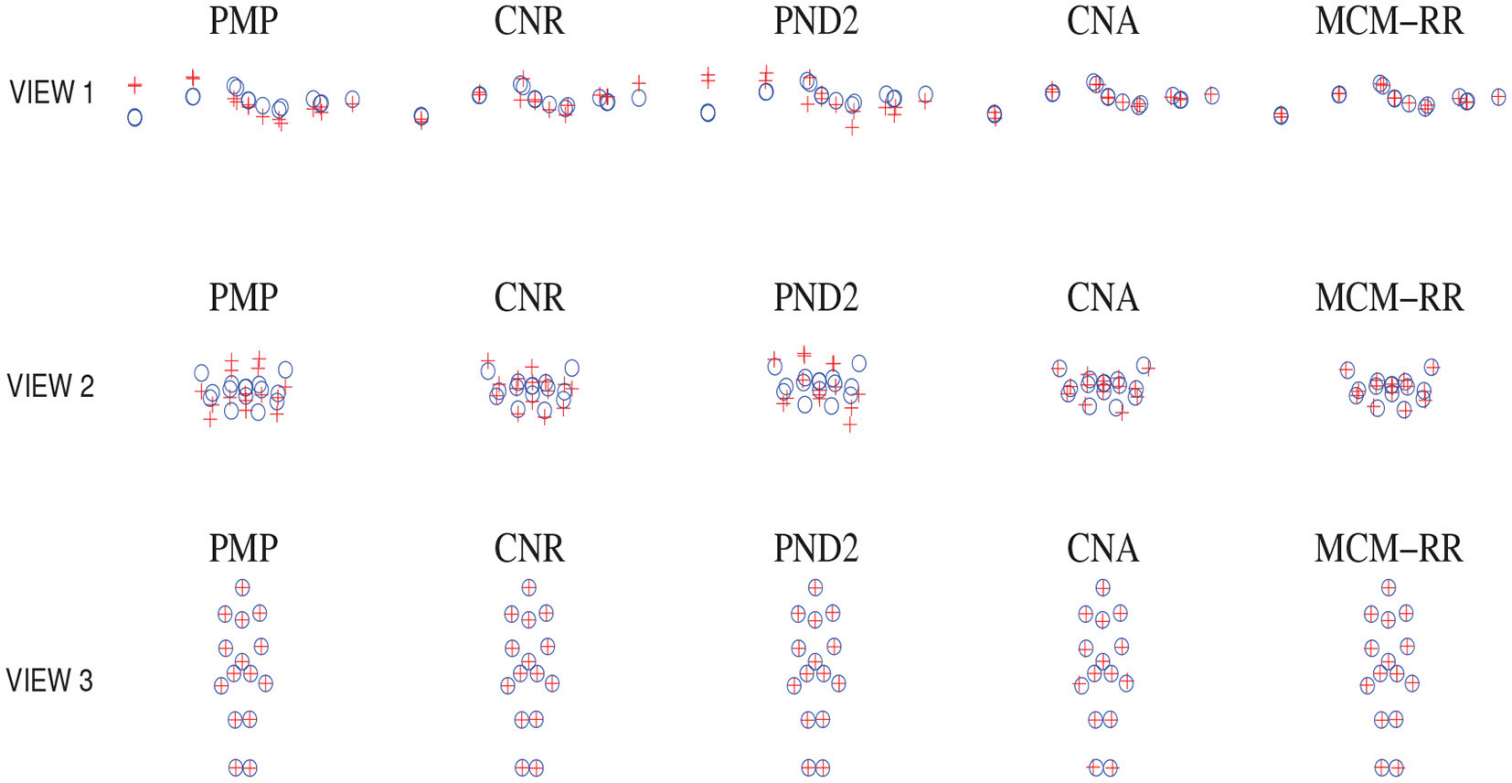
Comparisons of estimated shapes for single frame of *Jump* between MCM-RR and other methods from three different viewpoints. The symbol “°” denotes the observed real points, whereas “+” denotes reconstructed points.

Take one frame of *Jump* as an example. Figure 2 displays a comparison of reconstructed shapes between MCM-RR and the other methods from three different viewpoints. From Figure 2, we can see that compared to other methods, most estimated shapes of MCM-RR are closer to real points than that of the other methods.

### 3.2. Ablation experiment

In order to verify the feasibility of the proposed two strategies, the elastic net (denoted as CRA-EN) and similarity constraint (denoted as CRA-SC) are separately applied to the original algorithm CRA. Table 3 displays the mean and standard deviation (μ±σ) of 3D reconstruction errors ξ of eight motion categories for the four methods, respectively. Compared to CRA, both the elastic net and similarity constraint can decrease the 3D reconstruction errors. Therefore, the 3D reconstruction performance can be effectively improved once the two methods are simultaneously designed into CRA.

**Table 3:** Mean and standard deviation (μ±σ) of the 3D reconstruction errors ξ of eight motion categories for four methods.

**Sequence**	**CRA**	**CRA-EN**	**CRA-SC**	**MCM-RR**
Walk	38.98 ± 19.64	36.56 ± 19.18	38.64 ± 19.03	35.37 ± 18.49
Run	55.69 ± 18.13	52.60 ± 16.70	56.06 ± 18.03	52.64 ± 17.05
Jump	57.08 ± 41.56	46.61 ± 33.79	56.42 ± 39.52	44.56 ± 27.30
Climb	58.87 ± 24.73	49.99 ± 25.53	58.99 ± 24.88	50.25 ± 25.88
Box	72.90 ± 30.64	65.32 ± 27.64	73.02 ± 30.10	65.28 ± 26.82
Dance	102.36 ± 44.93	85.23 ± 35.63	101.49 ± 44.01	83.59 ± 34.88
Sit	75.68 ± 36.29	63.12 ± 26.79	74.92 ± 34.80	62.72 ± 26.79
Basketball	63.66 ± 27.92	57.81 ± 22.58	63.28 ± 28.29	57.57 ± 22.96

## 4. Conclusion

In this study, a multiple-constraint algorithm is devised to estimate the 3D shape of a 2D image sequence. Experimental results on the well-known CMU datasets demonstrated that the proposed methods have higher accuracies and more robustness. Compared with CRA, the 3D reconstruction error is decreased by at least 5.48%.

## Data availability statement

The datasets used in this article is from a public datesets, and it can be found in the CMU Graphics Lab Motion Capture Database.

## Author contributions

XC proposed the initial research idea, conducted the experiments, and wrote the manuscript. Z-LS supervised the work and advised the entire research process. YZ collected the dataset, analyzed the formal, and revised the manuscript. All authors reviewed and approved the final manuscript.
